# An overview on *Leishmania* vaccines: A narrative review article

**Published:** 2015-03-15

**Authors:** Hossein Rezvan, Mohammad Moafi

**Affiliations:** *Department of Laboratory Sciences, Faculty of Para-Veterinary Sciences, Bu-Ali Sina University, Hamedan, Iran****.***

**Keywords:** Adjuvant, Leishmaniasis, Vaccines

## Abstract

Leishmaniasis is one of the major health problems and categorized as a class I disease (emerging and uncontrolled) by World Health Organization (WHO), causing highly significant morbidity and mortality. Indeed, more than 350 million individuals are at risk of *Leishmania *infection, and about 1.6 million new cases occur causing more than 50 thousands death annually. Because of the severe toxicity and drug resistance, present chemotherapy regimen against diverse forms of *Leishmania *infections is not totally worthwhile. However, sound immunity due to natural infection, implies that vigor cellular immunity against *Leishmania* parasites, via their live, attenuated or killed forms, can be developed in dogs and humans. Moreover, genetically conserved antigens (in most of *Leishmania* species), and components of sand fly saliva confer potential immunogenic molecules for *Leishmania* vaccination. Vaccines successes in animal studies and some clinical trials clearly justify more researches and investments illuminating opportunities in suitable vaccine designation.

## Introduction

Leishmaniasis is a vector-borne protozoan disease spread by female sand flies, second to* malaria* in its prevalence, and it is currently amongst the six endemic diseases considered as high priorities worldwide. 1^,^^2^  World Health Organization (WHO) clarifies that approximately 0.2 to 0.4 and 0.7 to 1.2 million cases of visceral leishmaniasis (VL) and cutaneous leishmaniasis (CL), respectively, occur each year, amongst a susceptible population of 350 million in 88 countries on five continents.^3^^-^^6^ The parasite is transmittable through the bite of two *Phlebotmine* genera (*Phlebotomus* in the old world, and *Lutzomyia *in the new world) in a zoonotic (from infected animals such as dogs or rodents) or anthroponotic model.^7^^-^^13^ 

Diagnosis is based on clinical criteria manifested in humans, histopathology of lesions, detection and isolation of parasites from the lesions through biopsy (either by microscopy or culture methods), employment of soluble *Leishmania *protein in enzyme-linked immunosorbent assay (ELISA) method, and analysis of the small subunit ribosomal RNA genes employing the polymerase chain reaction (PCR).^14^^-^^16^ The current treatment is based on chemotherapy which relies on administration of drugs with high expenditure of purchase and serious side effects such as, toxicity, poor compliance and rapid induction of resistance in endemic areas.^17^^-^^19^ 

Vaccination remains the most appropriate opportunity for the prevention and safe treatment of all forms of the disease so development of a safe, effective and affordable anti-*Leishmania* vaccine is one of the main global public-health priorities and remains the most promising approach.^20^^-^^23^ This article provides the latest information regarding saliva, first generation, second generating, third generation, and live vaccines, respectively. 

**1. Saliva vaccine.** Immunization of mice either with *Phlebotomus papatasi* saliva or plasmid DNA comprising genes of *P. papatasi *or *Lutzomyia longipalpis *suggested that salivary molecules could be investigated as components of a vaccine against Leishmaniasis, hence, a cross sectional descriptive study aiming characterization of the antibody response to the saliva of *P. papatasi *in people living in endemic areas of CL was carried out in Tunisia.^24^^,^^25^ The results showed that the salivary proteins triggered the production of various antibody isotypes. Moreover, the immunodominant antigen called PpSP30 was recognized by all IgG subclasses, whereas PpSP12 was not by IgG4.^   25 ^ In addition, scientists say that the dose of salivary gland extract (SGE) used in immunization can significantly affect cellular immune response. To elaborate, one of Balb/c inoculated once by SGE presented a substantial augmentation in IL-10 production, whereas, the other group inoculated three times by the same preparation showed an increase in IFN-γ production in the draining lymph nodes of the inoculated mice.^   26 ^

**2. First generation vaccines (Whole killed parasite or** ***Leishmania*** **fractions). **Vaccines composed of whole killed parasites have been administered in randomized clinical trials (RCT) for prophylactic purposes.^20^^,^^27^^-^^29^ However, the results of administration of these vaccines had been associated with inconsistent efficacy due to application of variable criteria such as adjuvant doses and rout of the vaccine administration. For example, Iranian researchers in Razi institute investigated efficacy of autoclaved *L. major *(ALM) different in their formulation. The mentioned investigations substantiated ALM plus *Bacillus Calmette Guerine *(BCG) could not promote immunity rather than* BCG* alone.^   27 ^ Nevertheless Soudi and colleagues showed that co-administration of *BCG* and *ALM *rectally induced protective type 1 immune responses against *L. major* infection in comparison with *ALM* alone.^   28 ^

In innovative approaches sub-nits of *Leishmania* antigens (such as membrane antigens of *L. donovani *promastigotes or LAg) with novel adjuvant such as monorhosphoryl Lipid A (MPL-A), a non-toxic derivative product of the lipopolysaccharide (LPS) of *Salmonella minnesota*, have showed better prophylaxis in comparison with whole killed *Leishmania* cells.^30^^-^^32^ For example, Ravindar *et al*. administered *L. donovani *promastigote antigens associated with *BCG*, MPL-A, and cationic liposome in *Leishmania* antigen vaccine formulations against murine VL as a comparative study. This survey resulted in high levels of protection in mice immunized with *BCG *+ LAg and MPL + LAg, though, the highest level of protection was exhibited by the liposomal Lag immunized group.^   30 ^ Moreover, another sub-unit antigen, fucose mannose ligand (FML) antigen, could be able to show 43.3% protection against VL in mice. Furthermore, Brazilian scientists documented that usage of the FML vaccine in dogs has induced 92.0% and 95.0% protection in naturally exposed vaccinated dogs.^   33 ^

**3. Second generation vaccines. **Most vaccine studies pursue the sub-unite vaccines comprising of recombinant proteins or poly-proteins produced by DNA cloning. Second generation of refined vaccines, such as recombinant proteins associated with adjuvant or expressed in other microbial vectors, showed more feasible application for mass vaccination.^27^^,^^34^ Recombinant nature of vaccines implies that they facilitate their approachability in large scale, and cost effective production. Moreover, responses derived upon second generation vaccines can be strengthened and refined by relevant adjuvant.^   34 ^ However, second generation vaccines with different adjuvant meet differing potencies and confinements.^   35 ^ In this part, a list of defined peptide vaccine approaches being under development is provided and their potential strengths and weaknesses is summarized*.*

**3.1. Surface expressed glycoprotein (gp63) or leishmanolysin. **Leishmania surface leishmaniolysin (gp63) is a surface expressed glycoprotein implementing catalytic activity as a metalloproteinase which may protect *Leishmania* in macrophages.^36^^,^^37^ This protein was delivered by a plethora of immunization regimens, nonetheless, a suspicious discoveries from animal models were continued by mostly negative T cell responses in humans.^29^^,^^38^^,^^39^ For example, immunogenicity of gp63 subunit vaccine was analyzed either in its native or recombinant forms. Human T lymphocyte responses to gp63 derived from *L. amazonensis *(in its native or recombinant form) were evaluated in individuals with active or cured CL, mucocutaneus leishmaniasis (MCL) or VL. In this study it was substantiated that delivering of native gp63 failed to elicit the proliferation of T cell responses, whilst recombinant gp63 (rgp63) produced in *Escherichia coli *(*E. coli*) accomplished in T cell line stimulation. As a result, it was documented that rgp63 was efficient for human T cell elicitation in patients with active or cured *Leishmania *infection.^   40 ^ On the contrary in one another study it was substantiated, peripheral blood leucocytes (PBL) neither proliferated nor produced any IFN-γ following *in vitro* stimulation with the gp63, nonetheless, it could be an eligible safe candidate in the presence of appropriate adjuvant.^   33 ^

In recent years, liposome application, as potent adjuvant, in gp63 *Leishmania* vaccine was analyzed in new studies. For example, it was authenticated that distearoyl-phosphatidylcholine (DSPC) liposome used as vaccine adjuvant with the immunodominant gp63 of *L. donovani *promastigotes were able to promote significant protection against progressive VL in susceptible Balb/c mice. To paraphrase, control of disease progression and parasitic burden in mice vaccinated with gp63 in cationic DSPC liposomes was promoted via IFN-γ augmentation and down regulation of IL-4. In addition, CD8 T-cell responses was elicited by gp63 protein associated by DSPC.^   41 ^

In another study, the administration of rgp63-based vaccines derived from *L. donovani *with MPL-A (in a cationic liposome) has resulted in an increased number of IFN- γ producing effector T cells.^   42 ^

Furthermore, scientists has evaluated the protective and durable immunity of gp63 protein cloned into mammalian expressive vector (pcDNA3.1) by strategies comprised of DNA/DNA, DNA prime/protein-boost, protein/protein in the susceptible Balb/c mice against experimental VL using CpG-ODN as adjuvant. The researchers substantiated that vaccination based on gp63 DNA elicited immune responses and conferred protection.^   43 ^

In a new approach, Rezvan *et al*. analyzed immuno-genic peptides derived from *L. mexicana* in HLA-A2.1 transgenic (HHDII) and Balb/c mouse models. They have shown that none of the peptides predicted for Balb/c mouse MHC class I elicited CTL activity or significantly up-regulated the IFN-γ.^   44 ^ However, the immunogenicity of peptides derived from gp63 and restricted to HLA-DR1 was evaluated by FVB/N-DR1 transgenic mice model. The scientists evidenced that AAR peptides, which is derived from gp63 and restricted to HLA-DR1, can be a potent candidate in *Leishmania* infections due to its ability in IFN-γ and TH-1induction.^   45 ^

**3.2.** **Leishmania** **homologue for receptors of activated C kinase (LACK). **LACK protein belongs to a large family of WD 40 repeat (a short structural motif of approximately forty amino acids) proteins confined to eukaryotes. LACK gene expressed by both the promastigote and amastigote forms demonstrated polymorphic characteristic making it an appropriate molecule for genotyping *Leishmania* strains.^27^^,^^46^ In addition, LACK could be exploited along with other proteins (such asA2, NH, and K39) in ELISA with amended sensitivity.^   47 ^

Recently, considerable interest has been focused on LACK antigen as a potential vaccine candidate for leishmaniasis which is a result of its immuno-pathogenic role in murine *L. major *infection. To elaborate, it is authenticated that LACK antigen is potenttoenhanceIL-10 production, but down regulate of IFN-γ production, nevertheless, vaccination with recombinant LACK (rLACK) antigen in the presence of recombinant IL-12 (rIL-12) evokes CD4^+^ T cell inducing protection in mice against *L. major* infection (This protection correlated with augmentation of IFN-γ and reduction of IL-4).^48^^,^^49^ In one another study, researchers analyzed the ability of *Lactococcus lactis *expressing LACK and IL-12 gene in *Leishmania* protection in Balb/c mice. These scientists demonstrated that the preceded live vaccine could expand antigen-specific multifunctional TH1 CD4^+^ and CD8^+^ T cells and a systemic LACK-specific TH1 immune response.^50^^,^^51^ Moreover, researchers of Molecular Center in Spain demonstrated that immunization with modified Vaccina Ankara Virus expressing LACK antigen(extracted from *L. infantum*) stimulated CD4^+^ and CD8^+^ effector T cells.^   52 ^

**3.3. Hydrophilic acylated surface proteins (HASP). **Family of HASP comprising extensive and variant amino acid repeats which not only is expressed at the plasma membrane of intracellular (amastigote) of all Old World *Leishmania* species, but also HASP is presented in infective extracellular stage of* L. major*. However, some sub-genus of *L. aviannia* have lost HASP genes.^   53 ^ On one hand, some important enigmas remained unsolved about this plasma membrane protein. To elaborate, not only the pathway of HASP secretion is mysterious, but also the exact biological function of this protein remains obscure.^   27 ^ On the other hand, some novel findings which might be useful for *Leishmania* vaccine designation has been revealed. For example, HASPB1 induced protection or immunity does not need an adjuvant. This seems reasonable to assume that its mechanism of immunity is similar to DNA vaccine.^53^^,^^54^ 

**3.4.** ***Leishmania*** **-derived recombinant poly-protein (Leish-111f) or LEISH-F1 **Currently, few products enrolled as second generation vaccine have entered randomized clinical trials (RCTs) or veterinary testing. Just a single product LEISH-F1 (formerly known as Leish-111f) being a fusion protein of three relatively *Leishmania* proteins formulated with MPL-SE, has entered phase II clinical human testing.^   55 ^ LEISH-F1 is a single poly-protein composed of three integrated molecules: *L. major *homologue of eukaryotic thiol-specific antioxidant (TSA), *L. major *stress-inducible protein-1 (LmSTI1) and the *L. braziliensis *elongation and initiation factor (LeIF).^54^^,^^56^^,^^57^ 

In one study, researchers analyzed the safety and immunogenicity of the LEISH-F1 plus MPL-SE adjuvant when the protein was used in combination with sodium stibogluconate for the treatment of both mucosal and cutaneous leishmaniasis (MCL and CL respectively). These scientists proved that most of volunteers demonstrated IgG antibody and T-cell responses being specific to the LEISH-F1 antigen four weeks after the last injection of vaccine.^58^^, ^^59^ 

In addition, different RCT substantiated that LEISH-F1 + MPL-SE vaccine induced IFN-γ production. These interventional studies demonstrated this formulation is safe and immunogenic in healthy individuals with and without history of previous infection with* L. donovani.*^   60 ^

Furthermore, LEISH-F1 application was not just associated with MPL. For example, Sakai *et al*. in Japan analyzed the above-mentioned antigen with cholera toxin as a different adjuvant. Their trial showed that intranasal immunization with LEISH-F1 augments IFN-γ production and protects mice from Leishmaniasis in *L. major* infestation.^   61 ^

**4. Third generation vaccines (Naked DNA vaccines). **Though recombinant protein-based vaccines have achieved some degrees of protection in mice and dogs, they have faced complicated problems in the process of getting marketing authorization particularly in human medicine due to indispensable need to an adjuvant. Naked DNA vaccines are extremely safe since they do not contain any pathogenic organism that may revert in virulence and they have also achieved considerable success especially by gene gun in rodents. Nonetheless, they have often been proved to be insufficient for providing protection in non-murine models.^62^^-^^64^ 

In order to increase their immunogenicity, hetero-logous prime-boost (HPB) strategy can be implemented for DNA vaccine amendment. This strategy selectively expands memory T cells being specific for the vaccine antigen. Prime-boost assays analyzed against *Leishmania *with the LACK antigen accomplished protection in mice, nonetheless, it only conferred moderate protection in dogs. On the contrary, prime-boost vaccination using cysteine proteinase A and cysteine proteinase B antigens was highly protective in both mice and dogs, as a rare successful DNA vaccine in non-murine models.^   65 ^

Furthermore, scientists profess that a cocktail of different conserved antigens would probably provide the best protection against the parasite. The Researchers of the Pasteur Institute demonstrated that mice vaccinated with a cocktail DNA vaccine encoding cysteine proteinases type I, II and III with solid lipid nanoparticles were protected successfully against *L. major *infection.^   66 ^ In one different study, Ahmed *et al*. analyzed the immunity of DNA vaccines encoding LACKp24, TSA, LmSTI1 and CPa in Balb/c mice. They demonstrated that the cocktail DNA vaccine succeeded optimal protection when low parasite dose administered in the dermis of the ear.^   67 ^

However, there are some critical impediments jeopardizing DNA vaccine promotion in clinical trials. For example, human cells may become cancerous due to insertion of foreign DNA into their genomes.^   33 ^

**5. Leishmanization and live-attenuated** ***Leishmania*** **. **The inoculation of live and virulent *L. major *leading to a single lesion is called leishmanization (LZ). The LZ lesion upon cure prevents future natural infection which might be numerous lesions in sites, which might be important in the aspect of cosmetic issues.^   68 ^

On one hand, CL usually produces a self-healing lesion. Although rarely the lesion remains for a long time. Thus, scientists are targeting to develop other strategies aiming to exploit live Leishman’s bodies whilst the probability of refractory *Leishmania* lesions could be excluded.^68^^,^^69^ 

On the other hand, live attenuated *Leishmania* vaccines have become an attractive field because it has been cleared that complete *Leishmania *cDNA expression library injected into mice is more protective than any sub-pools of the library plasmids or a subunit.

Up to this time, several targeted gene eliminations have been analyzed to develop *Leishmania-*attenuated vaccine strains against CL. For example, researchers have administered dihydrofolate reductase thymidylate synthase (DHFR-TS) knockout parasites derived from *L. major*. They professed that this vaccine can produce durable protection in mice but application of this vaccine was not successful in rhesus monkeys.^   70 ^ Moreover, scientists of Manitoba University demonstrated that *L. major* strains being deficient for phosphoglycan (PG) gene (lpg2-) were able to confer protection against virulent *L. major* challenge, whereas, these attenuated strains are not capable of inducing IFN-γ production.^   71 ^

Furthermore, the administration of live attenuated vaccine emerges as a promising vaccine strategy within the scope of VL. To demonstrate, scientists tested the ability of a *L. infantum *deletion mutant, lacking both HSP70-II alleles (ΔHSP70-II), to provide protection against *Leishmania* infection in Balb/c mice. This interventional study showed that the vaccine (ΔHSP70-II) would be safe as immune deficient SCID mice. Hamsters (*Mesocricetus auratus*) infected with mutant parasites did not develop any sign of pathology.^   72 ^

In addition scientists have exploited a novel radioactive strategy aiming at attenuated vaccine production.^   73 ^ For example, Ultraviolet-A radiation and psoralen compound were administered for production of viable *Leishmania* called killed but metabolically active *Leishmania* strain (KBMA).^   73 ^ The KBMA* Leishmania* strains derived from either *L. major*or *L. infantum *were developed into the amastigote form inside macrophages. Furthermore, splenocytes from the mice vaccinated with either live *L. infantum chagasi* or KBMA* L. infantum chagasi *displayed similar cytokine patterns *in vitro*. These results suggest that KBMA technology is a potentially safe and effective novel vaccine strategy against the intracellular protozoa.^   74 ^

In spite of the investigations aimed at artificially attenuated wild *Leishmania *spp., some researchers focused on naturally attenuated *Leishmania *strains. McCall *et al*. demonstrated that Immunization with a naturally attenuated cutaneous *L. donovani* isolated from Sri Lanka protected Balb/c mice against VL.^   75 ^

## Summary and conclusions

In order to implement a successfully proposed strategies and actions, a long-term *Leishmania *vaccine plan needs to be developed. Through this review article, it was clear that many scientists with various approaches are dedicated to implement researches that have efficacy against Leishmaniasis, nonetheless, it was also proved that under the conditions of this study the majority of tests performed were not accomplished for WHO validation. However, we may be getting closer to develop a safe and effective leishmaniasis vaccine(s) as a result of newer vaccine approaches, which have evolved from whole irradiated live parasites, or the use of defined antigens such as LEISH-F, either in the form DNA vaccines or microbial vectors.
